# A High-Coverage Epitope-Based Vaccine Design for EIAV Envelope Polyprotein Using an Immunoinformatic Approach

**DOI:** 10.3390/vetsci13030279

**Published:** 2026-03-17

**Authors:** Ernesto Garay, Alberto S. Garay, Carolina Veaute, Adriana Soutullo

**Affiliations:** 1 Department of Immuno-Oncology, Beckman Research Institute of City of Hope, Duarte, CA 91010, USA; ernegaray@gmail.com; 2 Molecular Modeling Laboratory, Facultad de Bioquímica y Ciencias Biológicas, Universidad Nacional del Litoral, Ciudad Universitaria, Santa Fe 3001, Argentina; sergio.alberto.garay@gmail.com; 3 Laboratory of Experimental Immunology, Facultad de Bioquímica y Ciencias Biológicas, Universidad Nacional del Litoral, Ciudad Universitaria, Santa Fe 3001, Argentina; adrianasoutullo@gmail.com

**Keywords:** bioinformatics, equine infectious anemia virus, env-glycoproteins, B and T-cell epitopes

## Abstract

Equine Infectious Anemia (EIA) is a retroviral disease of equids, for which there is no vaccine particularly adapted to American viral strains. In this work we searched for possible epitope regions for the surface proteins gp90 and gp45, rationally employing the latest available bioinformatics tools that constitute the state of the art in the field. We selected eight regions that contain numerous overlapping epitopes that have a high coverage amongst American viral strains and designed a chimeric envelope protein with those proteins fused in tandem as a novel vaccine candidate. In silico predictors were used to analyze chimeric protein physicochemical and immunogenic properties, as well as its allergenicity and toxicity. Protein structure was predicted and validated, and its ability to trigger cytotoxic immune responses was predicted by molecular docking to ELA alleles. The proposed sequence is predicted to be highly immunogenic and sets the base for a novel EIAV vaccine that could be used to protect against several American field strains.

## Simple Summary

Equine Infectious Anemia is a disease of horses and related animals that is caused by a virus that remains in the body for life and can lead to weakness, fever, and severe health problems. There is currently no vaccine specially designed to protect animals from the forms of this virus that circulate in the Americas. In this study, we used computer-based methods to search for parts of the virus surface proteins that are most likely to be recognized by the immune system and to stimulate protection. We identified several promising regions and combined them into a single artificial protein to serve as a new vaccine candidate. We then used advanced digital tools to evaluate its safety, stability, and potential to trigger a strong and protective immune response. The results suggest that this designed protein could help develop a new vaccine capable of protecting animals against many virus forms found in the region, which would support animal health, prevent economic losses, and benefit communities that depend on horse-related activities.

## 1. Introduction

The Equine Infectious Anemia Virus (EIAV), recently classified as Lentivirus “equinfane” (Equine Infectious Anemia), is a significant global threat to the equine industry. It is a member of the subfamily Orthoretrovirinae [1,2]. The virus is characterized by its ability to cause a persistent infection that fluctuates between acute febrile episodes and lifelong inapparent carrier stages. It exerts strict control over replication [3,4]. The envelope glycoproteins, the Surface Unit (SU) gp90, and the Transmembrane (TM) gp45 are of particular importance in this process, as they are the primary targets of the equine immune response [5,6]. Due to their high antigenic heterogeneity and essential roles in viral entry via the Receptor Lentiviral Equine 1 (ELR-1), these proteins are the most promising candidates for vaccine development [7,8,9,10,11].

Notwithstanding the high prevalence of the disease throughout the Americas, particularly in Mexico and South America [1], current disease control measures entail the euthanasia of seropositive horses by Coggins’ Test, resulting in substantial economic losses [12,13,14,15,16,17,18].

The majority of studies conducted for the purpose of designing a vaccine have been constrained to the identification of specific B- and T-cell epitopes in EIAV glycoproteins, subsequent to experimental or natural infection [19,20,21,22,23,24,25,26,27,28]. The protective efficacy of the vaccines can range from preventing disease to causing death due to EIAV replication activity [29]. The only attenuated vaccine that has been utilized to provide 85% protection against various natural EIAV strains, despite exhibiting a 32% envelope protein divergence, was developed in China and employed up to 1990 [10,30,31]. Chinese studies have indicated a potential correlation between the effectiveness of the subject strain and its diversity. This diversity has been primarily linked to envelope proteins, which appear to emulate the diversity of viral quasi-species during the inapparent stage [10,32]. Notwithstanding its predominantly protective nature, the practice of vaccination has been terminated due to its inability to differentiate between infected and vaccinated animals (DIVA).

In addition to considerations of immunogenicity, the design of EIAV vaccine candidates must take into account diagnostic compatibility. Current immunochemical diagnostic assays for EIAV routinely employ the capsid protein p26 as the target antigen, which enables reliable serological detection of infected animals. Consequently, the inclusion of p26-derived sequences in a vaccine formulation could interfere with disease surveillance by preventing the differentiation of vaccinated from naturally infected animals. In this context, the development of DIVA-compatible vaccine strategies constitutes a critical component of EIAV control programs. Accordingly, the present study focuses on the rational in silico design of a chimeric vaccine candidate exclusively based on envelope proteins gp90 and gp45. These proteins are not used in standard diagnostic tests and constitute major targets of virus-specific immune responses.

To overcome these limitations, new strategies must integrate both broad protective efficacy and diagnostic compatibility. Conventional studies have focused on individual B- and T-cell epitopes. However, the emergence of Reverse Vaccinology [33] and the subsequent growth in knowledge of the host immune response have led to novel insights in vaccine design using in silico epitope predictions. Immunoinformatics tools employ a variety of approaches, including the creation and management of databases, the definition of both structural and functional signatures, and the design and application of predictive tools. These tools are used to identify multiple epitopes. These epitopes are useful in the design of chimeric vaccines. These vaccines target conserved epitopes in rapidly mutating pathogens, such as EIAV [34,35,36].

The equine leukocyte antigen (ELA) system exhibits substantial genetic polymorphism, with marked variability among breeds and limited population-level characterization in many regions, including the Americas [37,38]. This diversity poses challenges for epitope-based vaccine development, as allele-restricted responses may compromise broad population coverage. Therefore, a rational design strategy aiming at the identification of conserved, high-coverage epitopes with predicted binding across multiple ELA alleles represents a practical approach to maximize applicability in genetically diverse equine populations.

In this study, we employed a rational in silico design to develop a novel chimeric vaccine candidate based exclusively on the prediction of B and T-cell epitopes on EIAV envelope proteins. In order to address the genetic diversity of EIAV in the Americas, our approach offers a DIVA-compatible vaccine framework. This framework accounts for both viral variation and the diversity of local horse breeds.

## 2. Materials and Methods

### 2.1. In Silico Design and Validation Workflow

**Figure 1:**
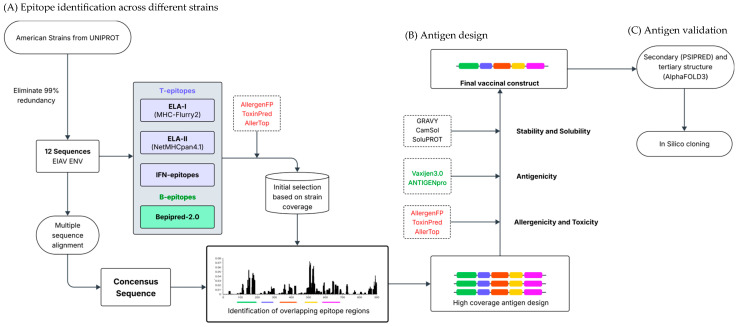
Flow chart showing the strategy followed to design a high coverage ENV based EIAV vaccine. (**A**) Epitope identification across different strains. ELA-I, ELA-II, IFN-γ and linear B epitopes were identified in 12 American EIAV ENV strain sequences, while allergenic and toxic peptides were discarded. Those regions with a higher number of overlapping epitopes and with the higher strain coverage were identified on a consensus sequence. (**B**) Antigen design. Three antigens containing the regions identified in A linked by different linker sequences were designed and analyzed through different predictors to select the one with the highest immunogenicity, solubility and stability, as well as the lowest allergenicity and toxicity. (**C**) Antigen validation. The secondary and tertiary structure of the final vaccinal construct antigen was predicted and validated, and its cellular CD8(+) T cell immunogenicity was predicted through docking of the higher scoring epitopes with ELA-I molecules.

The strategy used in this study to design a high-coverage EIAV vaccine based on the envelope (ENV) protein can be divided into three sections. The first section involved identifying conserved regions containing overlapping epitopes among different strains (Figure 1A). The second section involved assembling the conserved regions with different linkers and selecting the most appropriate one based on immunogenicity, stability, allergenicity, and toxicity (Figure 1B). The final section aimed to predict the antigen’s structure and validate the generated model (Figure 1C). The final step included a docking study of the most important CD8+ epitopes in the antigen with MHC class I molecules to predict the ability of the vaccine construct to induce cellular immune responses. Details of each step are provided below.

### 2.2. Retrieval of the Target Sequences and Multiple Sequence Alignment (MSA)

The target sequences of EIAV ENV polyprotein were retrieved from the UNIPROT database [39]. Given the objective of designing a vaccine that is primarily targeted towards American viral strains, only sequences originating from this continent were selected. Furthermore, the ENV polyprotein sequences exhibiting less than 99% identical residues were included in the analysis. To analyze the sequence similarity and obtain a consensus sequence among all the selected ones, the MAFFT program [40] was used with its default values, from the Jalview interface [41]. A consensus sequence was obtained from the MSA by placing the most frequent residue in each column.

### 2.3. Prediction of Protein Boundaries

PROCLEAVE software version 2020 [42] was utilized to predict potential enzymatic cleavage sites using both sequence and structural information. This software was run on the consensus sequence to establish the boundaries of each domain. In instances where feasible, the sites previously documented were incorporated.

### 2.4. Epitope Prediction

#### 2.4.1. CD8+ Epitope Prediction (ELA I)

The analysis was conducted using the MHCflurry-2.0 [43] program, which enables the prediction of pan-allelic peptides presented by ELA I (Equine Lymphocyte Antigen, equivalent to MHC-I). The model utilizes convolutional neural networks, a specialized form of deep learning architecture. The program was executed locally on all of the sequences selected for analysis. Among the epitopes identified (nine residues), those with a Presentation Percentile of less than 0.5% were retained. This threshold represents the acceptance of up to this value of false positives.

#### 2.4.2. CD4+ Epitope Prediction (ELA-II)

The predictions of MHC class II epitopes were conducted to identify 15-mer epitopes by means of the NetMHCpan-4.2 program [44], which was installed locally. The alleles for ELA-II (Equine Lymphocyte Antigen, equivalent to MHC-II) predictions were selected and downloaded from the Immuno Polymorphism Database (IPD) [45]. The program was executed locally on all of the sequences that had been selected for analysis. Peptides with a score_EL greater than 0.5 were utilized to identify the most probable binding epitopes.

#### 2.4.3. IFN-γ Inducing MHC Class II Binding Peptides

To identify peptides able to induce IFN-γ production by Th-cells, the “scan” program described by Dhanda et al. [46] was utilized. The IFNepitope web server (http://crdd.osdd.net/raghava/ifneepitope, accessed 3 February 2024) is capable of predicting IFN-γ induction with a maximum accuracy of 82.10% and of classifying epitopes as either those that generate IFN-γ or those that do not.

#### 2.4.4. Linear B-Cell Epitope Prediction

Linear B-cell epitope prediction was performed using the BEPIPRED-2.0 program [47] on the consensus sequence. Peptides with a score exceeding the 0.6 EL cutoff were selected to identify the most probable binding epitopes.

### 2.5. Criteria for Selection of Immunogenic Sequences

Taking the predicted epitopes from previous steps into account, regions containing overlapping epitopes recognized by ELA-I, ELA-II, and/or BCRs of B cells were defined. These regions were mostly within conserved domains. Each region contained at least two types of predicted epitopes. Additionally, epitopes within the principal neutralizing domain of gp90 associated with binding to ELR-1 were included.

To ensure optimal safety, we analyzed the allergenic properties of each region using a combination of three programs: AllerTOP v.2, AlgPred, and AllergenFP (https://www.ddg-pharmfac.net/AllerTOP/, https://webs.iiitd.edu.in/raghava/algpred2/, and https://ddg-pharmfac.net/AllergenFP/, respectively; accessed between 1 August 2024 and 25 September 2024). After eliminating allergenic epitopes, we checked their toxicity using ToxinPred2 (https://crdd.osdd.net/raghava/toxinpred/, accessed between 1 August 2024 and 25 September 2024).

Vaccine constructs containing overlapping epitope regions were designed with different amino acid linker sequences and were tested for allergenicity, toxicity, antigenicity, solubility, and stability. The allergenicity and toxicity were tested using AllerTOP v.2, AlgPred, and AllergenFP. The antigenicity was tested using VaxiJen 3.0 (https://www.ddg-pharmfac.net/vaxijen3/) and ANTIGENpro (https://scratch.proteomics.ics.uci.edu/explanation.html#ANTIGENpro). The solubility and stability were tested using GRAVY (https://web.expasy.org/protparam/), CamSol (https://www-vendruscolo.ch.cam.ac.uk/camsolmethod.html), and SOLUPROT (https://loschmidt.chemi.muni.cz/soluprot/). These servers were accessed between 1 August and 25 September 2024.

### 2.6. Proteasomal Cleavage Prediction

The immunoproteasome cleavage predictions of the EIAV multi-epitope construct with the linker EAAK were evaluated using the iPCPS server (http://imed.med.ucm.es/Tools/pcps/, accessed on 19 January 2026) in peptide mode 1 (nine amino acids), discarding those with internal cleavage sites and focusing on those with cleavage sites at the C-terminus.

### 2.7. Vaccinal Construct Structure Prediction

The secondary structure was predicted using PSIPRED 4.0 (http://bioinf.cs.ucl.ac.uk/psipred/) and the tertiary structure was predicted using the AlphaFold 3.0 server (https://alphafoldserver.com/v3.0.0/). The predicted Local Distance Difference Test (pLDDT) values were analyzed using the pLDDT scores stored in the mmCIF B-factor column. A predicted aligned error (PAE) matrix was created using the online PAE Viewer server (https://pae-viewer.uni-goettingen.de/). The structure was refined using GalaxyWEB (https://galaxy.seoklab.org/) and validated using SwissModel Structure Assessment Tools (https://swissmodel.expasy.org/assess). These servers were accessed between 3 October and 2 December 2024.

### 2.8. Assessment of Vaccinal Candidate Cellular Immunogenicity via Molecular Docking

Molecular docking experiments were performed to confirm that the selected epitopes could bind to their respective major histocompatibility complexes based on sequence alone, taking into account possible molecular receptor-epitope interactions.

We ran comparative molecular docking analyses for the four highest-scoring cytotoxic T lymphocyte (CTL) epitopes and their respective major histocompatibility complex class I (ELA-I) molecules. Each ELA-I 3D model was obtained via homology modeling, using the Equine MHC I crystal structure (PDB code: 4zuv) as a template. Five models were generated for each ELA-I variant using Modeller 9.21 software. The model with the lowest DOPE score was selected for each docking trial. Docking assays were performed using the GalaxyPepDock (version 2015) software. The complexes (ELAI-peptide) with the highest GalaxyPepDock scores were relaxed using the Relax application, and then the binding energy was estimated using the Interface Analyzer implemented in the Rosetta package.

## 3. Results

### 3.1. MSA and Definition of Consensus Sequence

**Figure 2:**
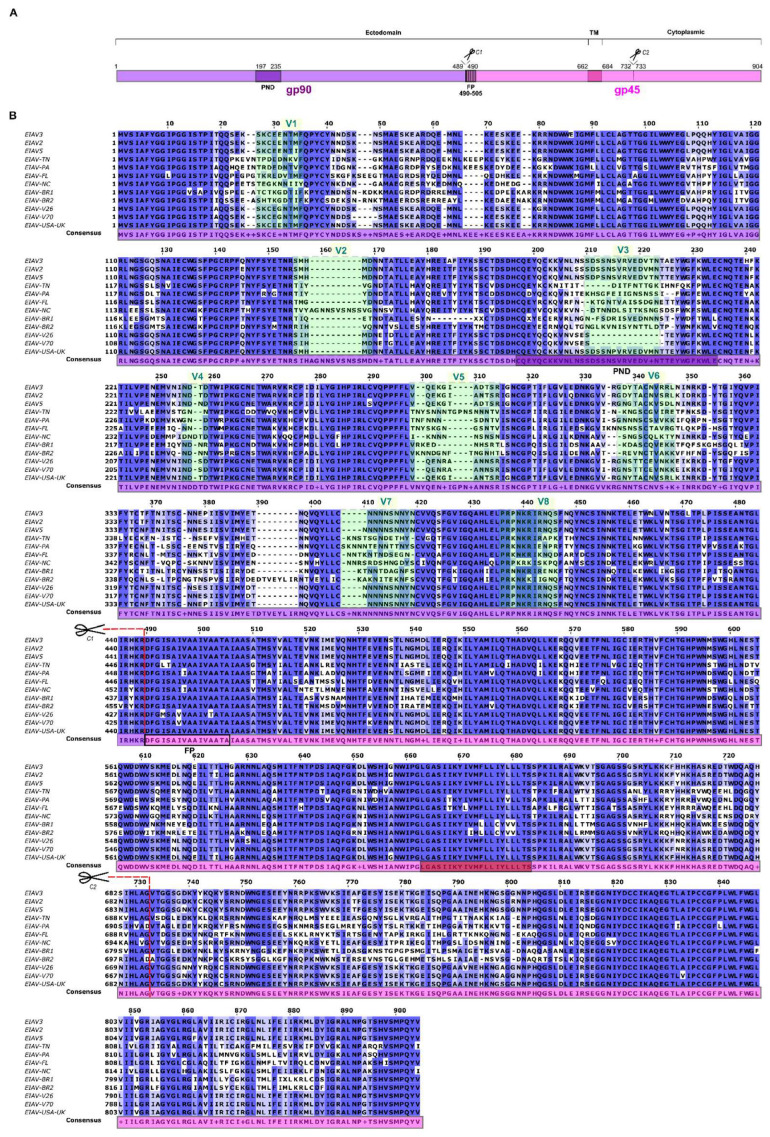
Overview of ENV protein sequences. (**A**) Schematic of ENV gp90 and gp45. The main regions of immature gp90 (purple, AA 1–489) and gp45 (pink, AA 490–904) are shown. Principal Neutralizing Domain (PND, AA 197–235) and the Fusion Peptide (FP, AA 490–505) are depicted in dark purple and vertical striped rectangles, respectively. Transmembrane domain (TM) in gp45 is shown as a dark pink rectangle (AA 663–683). C1 and C2 indicate predicted protein cleavage sites between residues 489–490 (Furin cleavage) and 732–733 (cathepsin B and a matrix Metalloprotease-2 cleavage, Table S1) respectively. (**B**) Multiple Sequence Alignment (MSA) of the ENV polyprotein of the American EIAV strains used in this work. The columns are colored in shades of blue according to their level of conservation. gp90 variable regions (V1–V8) are depicted as transparent green rectangles over the alignment. The features described in A are shown on the consensus sequence (PND, FP, TM, C1 and C2).

A total of 12 ENV polyprotein representative sequences of EIAV American strains were identified, and subsequently, these sequences were utilized to generate a multiple sequence alignment (MSA) (Figure 2). This approach was employed to visualize the conserved and variable regions between the sequences and to obtain a consensus sequence, which was subsequently used to design the high coverage vaccinal construct.

The less conserved sequences matched the variable regions of the gp90 protein (Figure 2, green squares), with the exception of two regions (AA 40–80 and 370–400). In contrast, the gp45 protein has been found to exhibit greater conservation, as has been previously reported [4,10,31,32]. However, region AA 760–800 in gp45 demonstrates a certain degree of variability.

The prediction of protease cleavage sites was conducted using Procleave software, and the identified sites were subsequently confirmed on the MSA. The furin cleavage sequence RHKR†DFGI (Figure 2, C1) was identified as one of the 20 highest-scoring sequences predicted by the program (Table S1). An additional potential cleavage site (HLAG–VTGG) was identified within the cytoplasmic tail of gp45, exhibiting a pattern of recognition consistent with that reported for cathepsin B and matrix metallopeptidase-2. This motif was included in the MSA (Figure 2, C2).

### 3.2. Epitope Prediction

**Figure 3:**
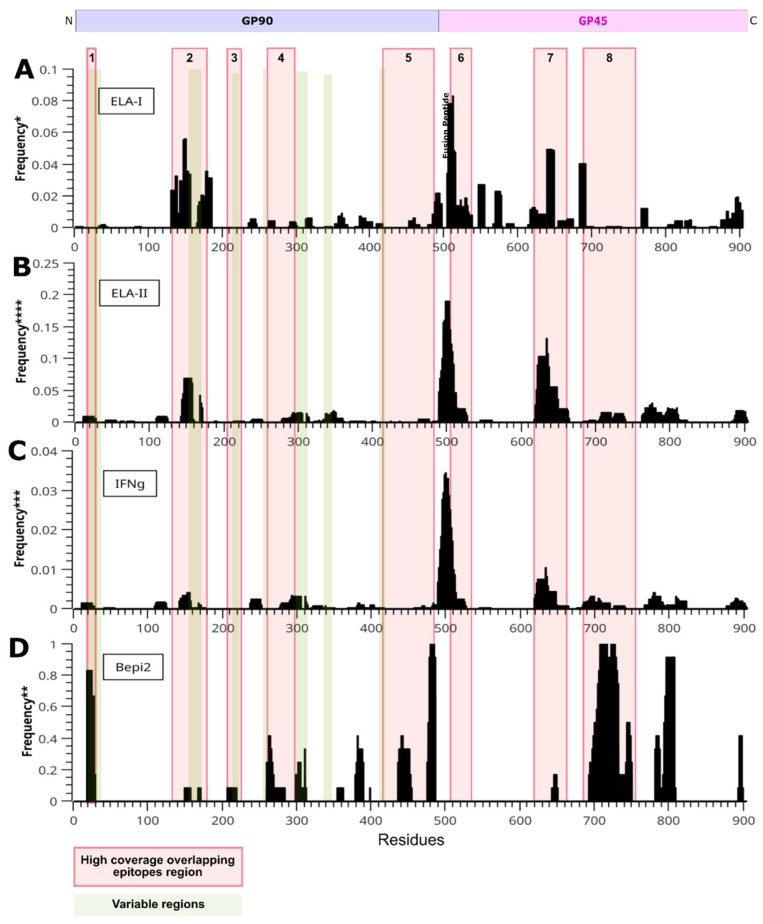
Epitopes predicted on the EIAV envelope protein. Bars represent the frequencies of each residue predicted as an epitope. To simplify each graph, only residues that exceeded the cutoff value for each prediction method were used for the statistics. Long green stripes represent the variable zones of gp90 and red ones indicate the high coverage overlapping epitopes regions found. (**A**) ELA-I epitopes predicted with MHCflurry-2.0 (Presentation Percentile < 0.5%) (**B**) ELA-II epitopes predicted by NetMHCpan-4.2 (EL > 0.5). (**C**) IFN-gamma inducing MHC class II binding peptides (**D**) Linear B-cell epitopes predicted by Bepipred (cut-off 0.6). * Frequency of residues predicted as ELA-I class antigen normalized by total number of epitopes. ** Frequency of residues predicted as linear B epitopes normalized by number of sequences. *** Frequency of residues predicted as IFN-gamma inducing epitopes normalized by number of sequences (12) and total number of positive results (247). **** Frequency of residues predicted as ELA-II class antigen normalized by the the number of sequences (12) and all possible combination of DQA or DRA (10) with DQB or DRB (31).

As the objective of this work was to design a high coverage vaccine for EIAV American strains, those epitopes that had a high representation across the different strains of interest were the main target to include in the design. With this objective in mind, all the 12 sequences were analyzed through different T and B epitope predictors. This resulted in a set of epitopes for each sequence which were then compared with those obtained for the other sequences and represented with a frequency number in Figure 3.

#### 3.2.1. CD8+ T-Cell Epitopes

MHC-Flurry2 was used with all the sequences described to identify ELA-I epitopes (Table S2). Three regions with a high frequency of ELA-I epitopes were identified. The first one in gp90, between residues 100 and190, the second one in the interface of gp90 and gp45 (residues 480–580) and the third one between residues 620 and 700, spanning a fragment of gp45 ectodomain and transmembrane region.

All twenty identified CTL epitopes have been recognized by the five Eqca-I alelles with high genotype frequency, Eqca 1*01:001; Eqca 16*01:001; Eqca16*03:001; Eqca 6*01:001; Eqca 7*02:001 and EqcaN*01:001.

#### 3.2.2. CD4+ T-Cell Epitopes

NetMHCpan4.2 was utilized with all the sequences described to identify ELA-II epitopes (see Table S3). It is noteworthy that five preliminary regions have been identified as exhibiting a high frequency of epitope occurrence (Figure 3B). The first sequence is located in the gp90 protein between residues 140 and 160, and the others are found in the gp45 protein in fragments 490–530, 620–660, 710–740, and 760–820.

A total of 34 helper T-cell epitopes were predicted within the analyzed sequences that exhibited broad reactivity across 24 of the 74 haplotype pairs. Specifically, the DQA1/DRB1, DQA1/DQB1, and DQA1/DQB2 loci exhibited a notably high gene frequency [37,38].

#### 3.2.3. IFN-γ Inducing MHC Class II Binding Peptides

A high frequency of IFN-γ epitopes was identified in specific regions, particularly at residues 140–160, 490–530, 620–660, 710–740, and 760–820. The aforementioned regions correspond to CD4+ high-frequency epitope regions (see Figure 3C), and they demonstrate 25 epitopes that are potentially associated with the secretion of IFN-γ.

#### 3.2.4. B-Cell Epitope Prediction

A total of six regions in gp90 protein with a high frequency of B-cell epitopes were identified: 20–30, 260–280, 300–310, 380–390, 435–455, and 475–490 (Figure 3D). For the gp45 protein, two regions localized to the intracytoplasmic domain have been found to have remarkably high scores between residues 690 and 750, and 780 and 810. In addition, three B-cell epitopes that are conserved among four distinct strains were identified within the PND region (AA 197–235). These epitopes have the potential to elicit neutralizing antibodies.

### 3.3. Selection of Polypeptide Sequences with Overlapping Epitopes

**Figure 4:**
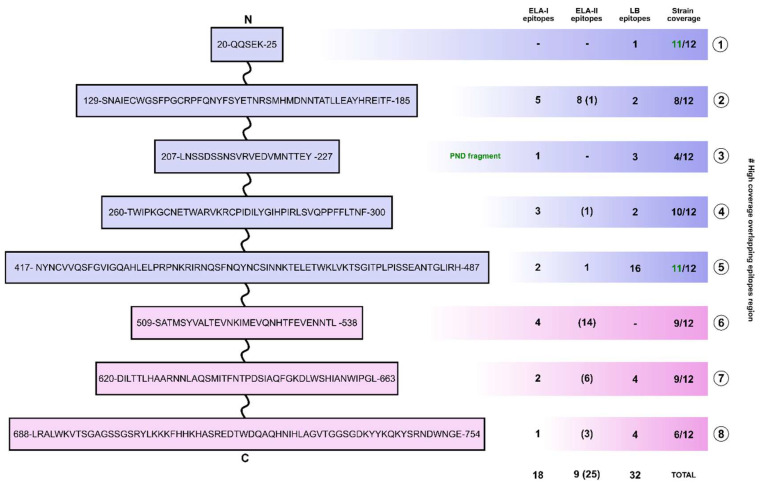
Preliminary immunogenicity summary of the high coverage overlapping epitope regions. Regionss are identified with numbers from 1 to 8 (right side), and their residues numbered according to the immature EIAV ENV consensus sequence. The number of Th1 epitopes is shown between brackets.

In order to optimize the size of the vaccinal construct, those regions with high frequency of overlapping epitopes, as well as a variety of epitope types, were selected to be included in the primary sequence. The study identified eight regions that were defined based on the presence of ELA-I and ELA-II recognized epitopes. These regions included those associated with the secretion of IFN-γ and B-cell epitopes (Figure 3, red boxes, Figure S2). Region 1 is an exception to the overall pattern, as it is distinguished by a high frequency of B-cell epitopes. Furthermore, region number 3 was incorporated due to its inclusion of the ELR-1 binding motif (AA 197–235). This domain is associated with the initial step of viral entry into cells and the primary neutralizing domain of the protein (see Figure 3 and Figure 4). As a general rule, gp90 variable residues were avoided (see Figure 3, green rectangles), although some were included due to the high frequency of the epitopes observed among different strains in those regions (regions 1 and 2).

The residues corresponding to the fusion peptide (AA 490–505) exhibit numerous overlapping epitopes at a high frequency among strains. However, chimeras incorporating this region exhibited the most unfavorable solubility and toxicity indexes (Table 1). Consequently, the FP was excluded from the design.

A synopsis of the selected regions and types of epitopes is presented in Figure 4, which illustrates a preliminary sequence from the N-terminal to the C-terminal end of the antigen sequence. Regions 1, 3, and 5 are predominantly B-epitope-enriched regions, exhibiting high coverage across diverse strains. The remaining five regions exhibit a higher prevalence of T-cell epitopes, featuring a diverse array of ELA-I, ELA-II, and IFN-γ triggering sequences, in addition to B-epitopes. According to the complete sequence, a total of 18 ELA-I epitopes, 9 ELA-II epitopes, 25 Th1 epitopes, and 32 B cell epitopes are identified.

To assess potential sequence similarity with host proteins, a tBLASTn analysis (NCBI) was carried out using the selected epitope sequences as queries against the core nucleotide database, restricted to Equus (Equus caballus). The search was performed with a word size of 5 and the BLOSUM45 substitution matrix. No significant alignments were identified using an E-value threshold of 0.1, indicating the absence of detectable homology between the predicted epitopes and equine nucleotide sequences.

### 3.4. Vaccinal Antigen Design and In Silico Characterization

#### 3.4.1. Linker Optimization

To facilitate the incorporation of the high-coverage, overlapping epitope regions delineated in Figure 4, a comprehensive analysis was conducted on four distinct linkers. This analysis encompassed the assessment of their contributions to protein stability, solubility, toxicity, allergenicity, and immunogenicity. A total of five distinct proteins were engineered by integrating each of the high-coverage, overlapping epitope regions with another one, though one copy of the evaluated linker (Table S4). Subsequent to this, the complete primary sequences were analyzed through a variety of predictors (Table 1). The first construct, which was designed with AAY linker, is predicted to be instable (instability index > 40), with low probability of adequate solubility (CamSol) and high probability of being an allergen (AllerTOPv.2). The second one, containing the flexible GGGGS linker, is also predicted to be instable (instability index > 40) and a probable allergen (AllergenFP). Conversely, the third construct containing the linker EAAAK exhibited satisfactory values across all the employed predictors, thereby being designated as the definitive antigenic sequence and designated as high coverage ENV (hcENV). The fourth and fifth proteins did not meet the established criteria for solubility and stability.

#### 3.4.2. Prediction of the Vaccinal Candidate Structure

**Figure 5:**
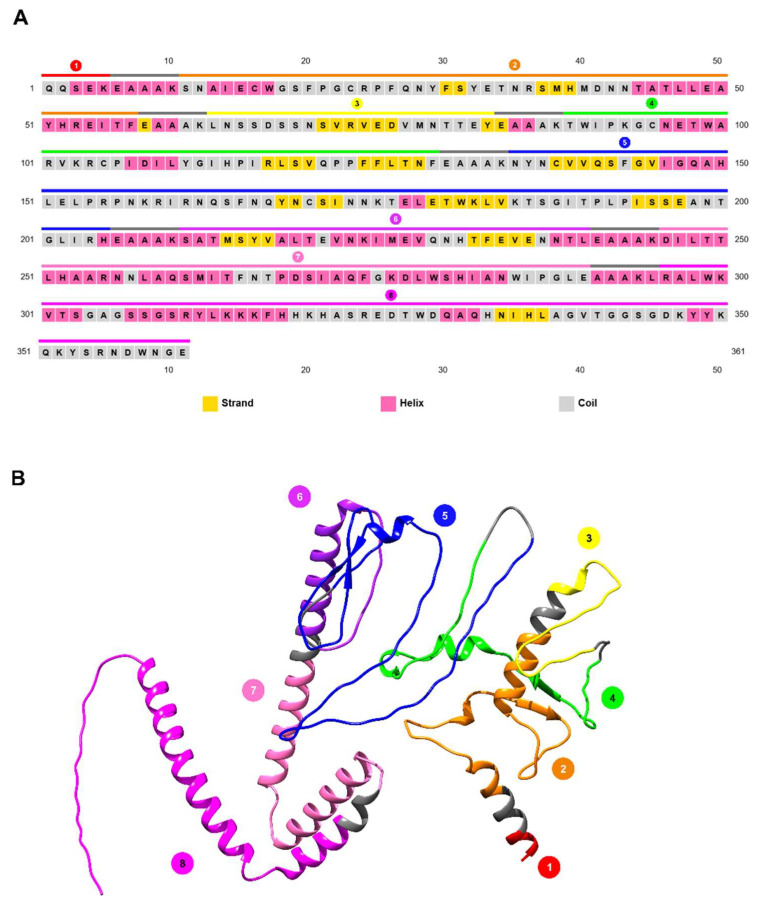
Predicted secondary and tertiary structures of hcENV construct. (**A**) Secondary structures as predicted by PSIPRED. High coverage regions are depicted in different colors on top of the sequence, while EAAAK linkers are shown in gray. Each residue is colored in terms of its predicted secondary structure: yellow for β-strand, pink for α-helix and gray for coils. (**B**) Predicted tertiary structure of hcENV. A 3D model was generated with AlphaFOLD 3.0, and the structure was refined with GalaxyWEB suite. High coverage regions are depicted in different colours, while EAAAK linkers are shown in gray.

The secondary structure of hcENV obtained with PSIPRED (Figure 5A) revealed that it is composed predominantly of random coils (46.5%), followed by α-helices (37.3%) and β-strands (15.8%). This finding indicates that the protein is moderately helical, with a significant proportion of disordered or flexible regions. A model of the predicted tertiary structure of the protein was obtained with AlphaFOLD 3.0 and refined with galaxyWEB (Figure 5B). A general inspection of the obtained model revealed higher percentages of α-helices and coil secondary structures (43.8% and 54.6%, respectively) in comparison with those obtained using PSIPRED, and a small percentage of residues forming β-strands (1.7%).

The short B epitope containing Region 1 (red) acquired an α-helix conformation, while Region 2 (orange) is composed of a larger α-helix, a small α-helix, and a β-strand connected by coils. The region 3 (yellow) is constituted by a short α-helix that is a continuation of the preceding EAAAK linker, while the remainder adopts a coil structure that extends into region 4 (green). Region 4 contains a β-strand that establishes a β-sheet conformation with region 2′s β-strand, as well as two small α-helix regions that are succeeded by a long coil structure. This extended coil comprises the concluding remnants of Region 4 and nearly the entirety of Region 5 (depicted in blue), with the exception of a diminutive α-helix and an intra-region β-sheet. Regions 6 (purple) and 7 (light pink) form a long α-helix, with the linker positioned centrally within the structure. The remaining protein comprises two moderately long α-helices that appear to interact with each other, as well as a final long α-helix, all of which are separated by short coil regions (the last two helices are part of Region 8, which is bright pink). A relatively long coil marks the C-terminal end of Region 8.

**Figure 6:**
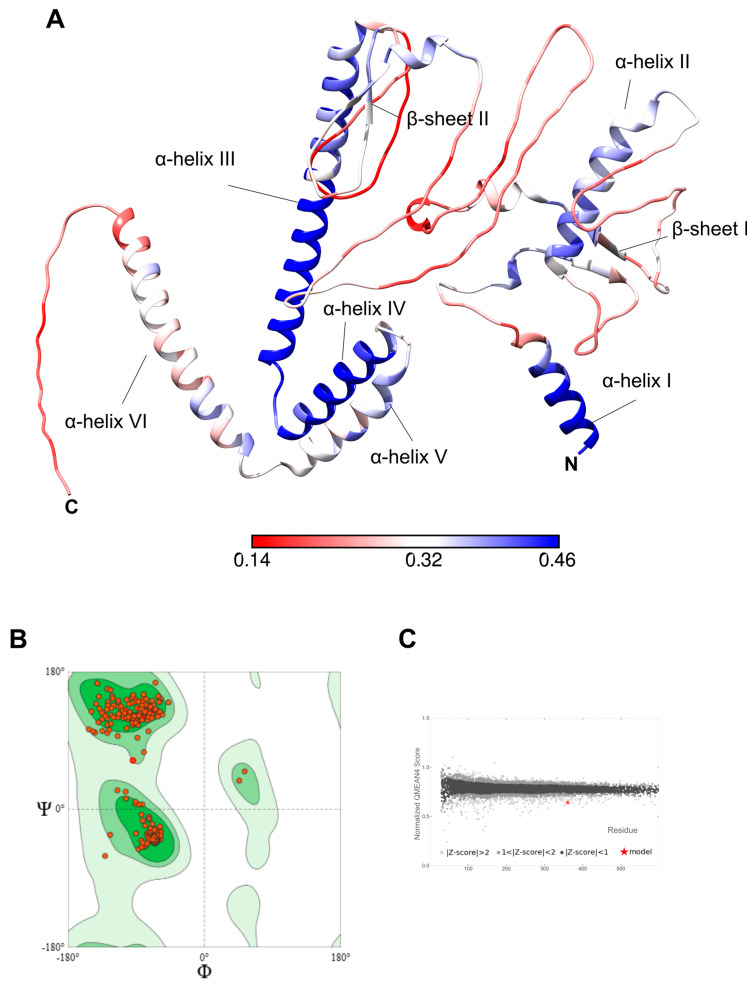
Assessment of the quality of the model. (**A**) Local quality estimate. hcENV 3D model was colored according to the QMEAN local score of each residue. Residues with lower QMEAN values are depicted in red (lower quality), while the ones with higher values are coloured in blue (higher quality). Color scale ranges from 0.14 to 0.46 QMEAN local score. (**B**) Ramachandran plot. (**C**) Normalized QMEAN value of hcENV model. The value obtained for the model is compared to a set of non-redundant Protein Data Bank structures.

The predicted structure of hcENV was analyzed with SwissModel, and the local quality of the model was assessed (Figure 6A). α-helices I to IV are the regions with the higher b-factor values, while helices V and VI have intermediate values, as well as β-sheets 1 and 2. Coil structures demonstrate the lowest degree of reliability among the model’s structures. Notwithstanding, the stereo-chemical quality of the model is very good, as 99.7% of the residues are located on the favored regions of the Ramachandran plot (Figure 6B). In addition, the normalized QMEAN value of the model is analogous to the experimentally solved structures of proteins of a similar size to hcENV (Figure 6C), suggesting that the model exhibits an acceptable quality.

Furthermore, the proteasomal processing of the EIAV multi-epitope construct was evaluated using the iPCPS server (http://imed.med.ucm.es/Tools/pcps/, accessed on 19 January 2026). The predicted cleavage pattern revealed no high-confidence internal cleavage sites within the EAAAK linkers, confirming their proteasome-resistant behavior. Multiple 9-mer peptides corresponding to predicted CD8+ T-cell epitopes were identified, with no evidence of internal degradation (Table S5).

### 3.5. Docking of CD8+ T-Cell Epitopes on ELA-I

**Figure 7:**
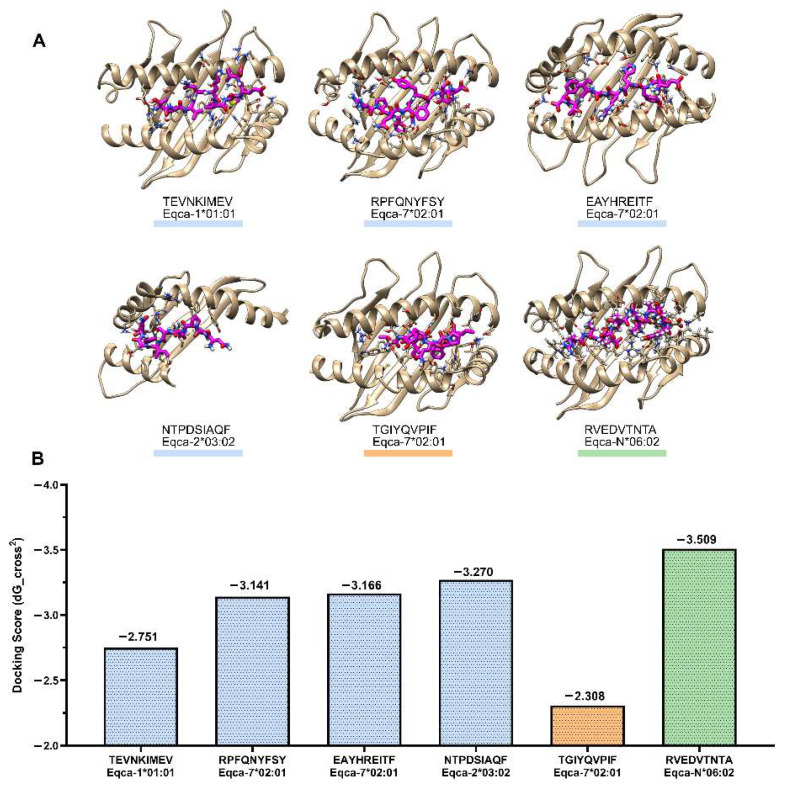
Docking of epitopes with ELA-I molecules. (**A**) Best structural results obtained for each docking experiment. Backbones of the ELA receptors are rendered as brown ribbons, with the side chains that interact with the epitope represented as sticks. The peptide epitopes are displayed as stick representation, with the carbon atoms colored in pink. All the other atoms are colored with CPK coloring scheme. With red dot lines are shown the hydrogen bonds formed between the receptor and the epitope. (**B**) Docking score (DG_cross) obtained for each peptide-ELA complex. Docking assays were performed using GalaxyPepDock (2015) software. Complexes (ELA-I-peptide) with the highest GalaxyPepDock score were relaxed using the Relax41 application, and then the binding energy was estimated using the Interface Analyzer42 implemented in the Rosetta package. As controls, a peptide with MHC-Flurry low percentile affinity score value (TGIYQVPIF, orange) and a crystallized solved ELA–epitope complex (PDB:4ZUV, RVEDVTNTA green) were included in the analysis.

Due to the significance of cytotoxic T lymphocyte responses in resolving early episodes of viraemia and clinical disease in EIAV-infected horses [19,21,22,24,48,49,50], hcENV representative ELA-I epitopes were subjected to docking experiments with ELA-I alleles (Figure 7A, Table S6).

All the studied ELA-I epitopes demonstrated a negative stabilizing score (Figure 7B), which was normalized to the interface area of each complex. In order to validate the method, a peptide with low percentile affinity [TGIYQVPIF] (356–405) was utilized. This peptide was expected to show the least favorable (least negative) score value. In addition, a crystallized solved ELA–epitope complex (PDB: 4ZUV, RVEDVTNTA) [51] was included. This complex was expected to give a very favorable (most negative) docking value.

The low percentile affinity peptide [TGIYQVPIF] (356–405) demonstrated a high docking score, despite the prediction of a low score by MHC-Flurry. The three epitopes of gp90 predicted by MHC-Flurry: [TEVNKIMEV] (518–526), [RPFQNYFSY] (142–150), [EAYHREITF] (177–185) and one of gp45: [NTPDSIAQF] (640–648) exhibited higher docking scores compared to the lowest value observed for the low-scoring reference. However, these values were lower than the highest value recorded for the experimental complex. [RPFQNYFSY] (142–150) and [EAYHREITF] (177–185) interactions showed high score (ddG) values, and are stabilized by numerous hydrogen bonds, including several specific ones with the side chains. The [TEVNKIMEV] (518–526) epitope of gp90 showed the lowest docking value.

It is noteworthy that the complex of [NTPDSIAQF] (640–648) with Eqca-2*03:02 exhibited a favorable score, despite the limited solvent accessible surface of the complex interface (Figure 7A). This is attributable to the fact that Eqca-2*03:02 possesses an N-terminal helix that is shorter in comparison to that of other ELA-I molecules.

## 4. Discussion

In this study, we applied the principles of Reverse and Structural Vaccinology [35] to rationally design a multi-epitope vaccine against American strains of the Equine Infectious Anemia Virus (EIAV) based on fully sequenced Env genes. Our methodology integrated epitope prediction tools with rigorous assessments of solubility, antigenicity, allergenicity, and toxicity.

The envelope proteins (gp90 and gp45) were selected for analysis as they are primary determinants of lentivirus vaccine efficacy, despite their diversity [15,52,53]. A multiple sequence alignment was utilized to identify conserved and variable regions across strains:

The variable regions were primarily localized within gp90 protein. Specifically, the Principal Neutralizing Domain (PND) in the V3 region was included, in spite of exhibited low sequence similarity consistent with previous reports [54,55]. On the other hand, gp45 protein is more conserved, aligning with established literature, being the Fusion Peptide (FP), and transmembrane domain showed high conservation [4,10,31,32].

Furthermore, protein cleavage site predictions successfully localized the gp90–gp45 boundary at a furin cleavage site, matching earlier descriptions [56]. We also identified a potential cleavage motif within the cytoplasmic tail (CT) of gp45, in a region previously linked to viral protease activity [57]. While cathepsin B has been implicated in similar contexts, the role of Matrix Metallopeptidasee-2 in EIAV gp45 processing has not yet been documented. Given the sequence context and predicted accessibility, we propose this cleavage as a plausible hypothesis for gp45 processing. However, the specific protease and its biological relevance require experimental validation. Current evidence suggests that truncation of the gp45 C- terminus significantly impacts protein trafficking, Env precursor cleavage, and overall virion production [58].

In this study, epitope analysis targeted ELA-I, ELA-II, IFN-γ, and B-cell motifs due to their critical role in equine anti-viral immunity. Others authors extensively screened both ENV proteins to identify conserved epitopes shared across multiple strains [21,49,50,52].

We predicted five MHC-I epitopes within residues 129–185 that are recognized by 15 MHC-I alleles. Furthermore, we identified two CTL epitopes at the C-terminal end of this region (142-RPFQNYFSY-150 and 177-EAYHREITF-185). To our knowledge, this represents the first report of CTL epitopes localized within this specific domain of the EIAV envelope [21,49,50,52], highlighting a novel target for inducing cellular immunity against American EIAV strains. Notably, the CTL epitope (142–150) exhibited the highest affinity for the Eqca-7*02:01 allele. From a clinical perspective, the high frequency of this allele in horse populations [59] is a crucial finding; it suggests that a vaccine incorporating this epitope could provide broad-spectrum protection across a significant percentage of the equine population. This computational evidence aligns with our previous experimental data, where the synthetic peptide gp90-B (130-NAIECWGSFPGCRPFQNYFSYETNRSMHMDNNTATLLEAYHR-171) was recognized by ELA-I molecules in 4 out of 5 unapparent carrier horses [59], further validating its immunogenic potential in a natural infection context.

One of the earliest characterized immunodominant CD8+ T-cell epitopes in the gp90 protein is Env-RW12 [RVEDVTNTAEYW], located within the V3 region [19,49,50]. In the corresponding 204–228 region, we identified a CTL epitope, [217-RVEDVTNTTEY-227], recognized by four ELA-I alleles. This sequence is highly analogous to the epitope previously crystallized in complex with the ELA receptor (PDB: 4ZUV). However, our docking simulations (Table S5) indicated that the substitution of two amino acid residues was sufficient to reduce its predicted binding affinity compared to the reference. Despite this predicted decrease in binding capacity, Tagmyer et al. [49] identified a protective synthetic peptide [SNPV-RVEDVMNTTEY-WGFKW] (213–232) that correlates with robust Env-specific CTL responses [11,39,43]. Given this substantial experimental evidence, we opted to include the [RVEDVTNTTEY] epitope in our design, prioritizing its proven biological relevance over its lower in silico binding scores.

Furthermore, we predicted three CD8+ T-cell epitopes recognized by eight ELA-I alleles within the 271–300 region. These sequences were within into the peptides [KRCPIDILYGIHPIRLCVQPDCTL] (273–296) and [IHPIRLCVQPPFFLVQEKGI] (283–302), both of which have been previously associated with protective CTL immune responses [21,59].

We identified two additional ELA-I epitopes with significant immunogenic potential in the 417–487 region of the gp90 glycoprotein. The first (417–425) is nested within the synthetic peptide [NNYNCVVQSF-GVIGQAHLEL] (416–435), which has been previously linked to protective CTL responses [21,49]. The second epitope (472–480) is localized within the gp90-A [ETWKLVKTSGI-TPLPISSEA-NTGLIRHKR] (461–489) synthetic peptide. This region is of particular interest as our earlier studies demonstrated its dual recognition by both ELA-I and ELA-II molecules [59]. The ability of this domain to engage both MHC classes suggests it may play a pivotal role in eliciting a coordinated cellular immune response, bridging CD4+ helper and CD8+ cytotoxic T-cell activity.

While the identification of these epitopes in gp90 addresses the challenge of inducing targeted cellular responses against the most variable regions of the virus, a truly comprehensive vaccine must also leverage the structural stability of the transmembrane protein. Consequently, we extended our analysis to gp45, seeking conserved domains that could provide broad-spectrum protection and enhance the overall structural integrity of the multiepitope construct.

In the 490–538 region of the gp45 protein, we identified six ELA-I epitopes. The first two exhibit significant overlap (residues 504–512) and are predicted to be presented by fifteen different allotypes. The remaining four epitopes, located at the C-terminal end of this domain, were recognized by all analyzed alleles except for Eqca 2*01:01. To our knowledge, this constitutes the first report of potential ELA-I epitopes within this specific region [15,21,49,50,52].

Additionally, in the 620–663 region, two ELA-I epitopes were predicted. Notably, [640-NTPDSIAQF-648] was recognized by 12 allotypes with high affinity percentiles and robust molecular docking scores, particularly with Eqca-2*03:02 alleles. The second epitope, [655-HIANWIPGL-663], was recognized by eight allotypes. A similar sequence, [IGNWIPGL-GASSIKYIMFL] (656–674), was previously reported to induce a CTL response in only two of twelve horses immunized with the EIAV D9 vaccine; consequently, it was not initially classified as a protective CTL epitope [60]. However, our findings suggest that its immunogenicity may be allele-dependent.

The final CTL epitope identified (722–730) is nested within the gp45-B (696–715) synthetic peptide sequence and is presented by ELA-I molecules, consistent with previous experimental observations [59,60].

While numerous synthetic peptides from both EIAV envelope proteins have yielded positive results in CTL assays, only eight have been definitively associated with a protective response [15,21,50,59]. Remarkably, four of these protective peptides share identical or highly similar sequences with the ELA-I epitopes predicted by our bioinformatic analysis. Furthermore, all twenty epitopes described in this work are predicted to bind ELA-I alleles with high genotype frequencies, such as those previously identified in Thoroughbred populations [61], reinforcing the by potential for broad vaccine coverage.

The protective effect of IFN-γ production by CTL and Th cells was described for the attenuated vaccine EIAV DLV121 [10,20,49,60,62]. Here, we predicted Th cell epitopes able to bind to high frequency ELA-II [37,38], and potentially associated with IFN-γ induction.

Among the defined candidate regions, the 129–185 domain contains eight overlapping Th-cell epitopes recognized by 34 ELA-II allele pairs. These computational findings are in strong agreement with previous experimental data obtained from the [LNGSGQS-NAIECWGSFPGCR] (123–142) and gp90-B (130–171) peptides, validating the immunogenic relevance of this region [23].

A second region highly enriched in Th-cell epitopes spans residues 490–526. These epitopes are predicted to be associated with IFN-γ production and are potentially presented by 42 ELA-II allele pairs. To date, experimental data regarding this specific region remain scarce; however, our results suggest that its inclusion in a multiepitope vaccine is highly warranted to enhance cellular immunity.

Another promising domain comprises residues 620–663, which harbors six overlapping Th1 epitopes with broad recognition across 32 allele pairs, showing a high affinity for DQA alleles. This is consistent with the findings of Tagmyer et al., who identified two peptides, named 60 [MITFNTPDSIAQFGKDLWS] (636–654) and 61 [AQFGKDLWSHIGNWIPGLGA] (646–665), that were recognized by DQA alleles and associated with protection against experimental infection with both homologous and heterologous strains [21,49].

Achieving sterile immunity against lentiviral challenges requires a synergistic immune response, integrating both robust CTL activity and the induction of broadly neutralizing antibodies (bNAbs). A primary objective in lentiviral vaccine development, particularly in HIV-1 research, is the elicitation of bNAbs capable of neutralizing a diverse array of viral strains [63]. Although predicting these responses remains a significant challenge, a vaccine design that incorporates multiple conserved epitopes and domains involved in virion-receptor interactions represents a highly promising strategy [64,65]. The regions selected in this study were specifically chosen to align with this multifaceted approach. Moreover, the selection of both the vaccine platform and the adjuvant is imperative in order to delineate the immune profile. The utilization of nucleic acid-based vaccines, in conjunction with formulations comprising lipid-based particles, has been shown to elicit Th1 and CTL responses, which are effective in neutralizing viral infections [35].

Furthermore, we analyzed linear B-cell epitopes within both envelope glycoproteins. Our results identified 32 linear B-cell epitopes that co-localize with predicted ELA-I and/or ELA-II motifs. The identification of these overlapping immunodominant regions is significant, as it suggests the potential for a highly integrated immune response. Notably, many of these epitopes are consistent with previously documented experimental findings, further reinforcing the biological validity of our multiepitope construct [5,6,25,26,28,66].

We identified two B-cell epitopes that overlap with T-cell motifs within the 129–185 region. This finding is consistent with experimental evidence showing that antibodies from infected horses recognize specific peptides in this domain, including [WGSFPGC-RPFQNYFSYET] (135–152), [SYETNRSMHMDN-NTATLLEAYHREITF] (149–185), and [NAIECWGSFPGC-RPFQNYFSYETNRSMHMDN-NTATLLEAYHR] (130–171) [5].

The 207–227 region presents particularly compelling features, as it encompasses part of the Principal Neutralizing Domain (PND, 197–235) and the specific sequence involved in the interaction with the ELR-1 receptor (212–223) [3,38,46]. However, this region poses two potential challenges: its inherent sequence variability and the presence of a potentially glycosylated asparagine identified in all twelve sequences studied (Figure S3). While N-glycosylation is often viewed as a viral immune evasion mechanism that sterically shields surface proteins, substantial evidence suggests that glycan structures can actually enhance the antigenicity and immunogenicity of neutralizing epitopes. Predicting these effects remains complex, as glycosylation has been shown to both improve or diminish antibody neutralizing activity. For instance, both glycosylated and non-glycosylated peptides against HTLV-1 gp46 were highly immunogenic in rabbits, eliciting high-titer antibodies capable of binding the native protein [67]. In contrast, the mutation of N-linked glycosylation sites in the Ebola virus GP2 proved detrimental to antigenicity and immunogenicity, likely due to conformational disruptions [68]. Conversely, strategic glycan masking in SARS-CoV-2 and other viruses has demonstrated that modifying glycosylation can focus the immune response toward conserved, protective regions, possibly by unmasking epitopes [69]. The choice of vaccine platform (e.g., bacterially expressed recombinant proteins, nucleic acids, or viral vectors) is critical here, as it dictates the final glycosylation state of the immunogen. Therefore, we justify the retention of these experimentally validated neutralizing regions despite their potential glycosylation sites; these glycans may be essential for maintaining the native epitope architecture necessary to induce protective antibodies relevant to the wild-type virus.

Most of the predicted B-cell epitopes were concentrated within the 417–487 region, aligning with experimental data that demonstrates high reactivity in the C-terminal residues of gp90 [28,66]. Additionally, the eighth region (residues 688–754) harbors four B-cell epitopes, consistent with previous reports [6]. Although this domain includes part of the cytoplasmic tail (residues 680–820), a region traditionally considered less accessible for antibody induction, the presence of specific antibodies in infected horses suggests it is indeed exposed during natural infection. While its role in protection remains to be fully elucidated, its immunogenicity is clearly documented [6]. Additionally, given the presence of four T-cell epitopes, inclusion of this region is warranted.

A critical consideration in our vaccine design is antibody-dependent enhancement (ADE), a phenomenon where certain antibodies facilitate viral entry into host cells, such as macrophages. Previous studies have shown that immunization with the full-length recombinant gp90 of EIAV can paradoxically lead to a severe enhancement of viral infection [70]. Consequently, utilizing the entire gp90 sequence is contraindicated. Instead, we propose the use of selected immunogenic domains as a strategic alternative to mitigate ADE risks. This approach mirrors successful vaccination strategies developed for other viruses, such as MERS-CoV, where the use of discrete domains rather than the full-length spike protein effectively eliminated ADE [71]. Furthermore, minimizing ADE risk may involve the targeted induction of IgG2/5/6 isotypes, which exhibit reduced binding affinity to the FcγRII/III receptors present on equine monocytes and macrophages [71]. By focusing on these specific regions, our multiepitope construct aims to elicit a protective response rather than enhancing the viral infection.

The integrative strategy employed in this study facilitated the identification of 75 T- and B-cell epitopes, which were systematically clustered into eight candidate immunodominant regions: five within gp90 and three within gp45. Notably, three of the linear B-cell (LB) epitopes are localized within the Principal Neutralizing Domain (PND). Despite the extreme sequence variability characteristic of this domain, these epitopes demonstrate the potential to induce antibodies across 4 of the 12 strains analyzed—a significant finding for broad-spectrum vaccine design.

To assemble these regions into a functional multiepitope construct, we designed a set of fusion proteins using a tandem arrangement joined by specialized linkers (EAAAK, AAY, and GGGGS), each selected for its specific structural or processing role [72,73,74,75,76]. The final vaccine candidate (hcENV) was proposed using EAAK linkers, and its complete sequence was subjected to secondary and tertiary structure modeling, adopting a globular conformation characterized by rigid helices separated by flexible regions. The strategic use of EAAAK linkers represents a calculated compromise between structural compartmentalization and antigen processing. The use of EAAAK linkers represents a compromise between structural separation and antigen processing. While not specifically optimized to enhance proteasomal cleavage, EAAAK can limit undesired internal degradation and preserve epitope boundaries, which is particularly advantageous in vaccine constructs designed to activate CD8+ T-cell responses. Consequently, the regions predicted to be rigid and solvent-exposed are positioned to efficiently trigger B-cell responses, while the interspersed flexible regions are expected to allow for effective intracellular processing and MHC presentation of T-cell epitopes, resulting in a balanced overall immunogenicity [77]. Although antibody affinity maturation and neutralizing potency cannot be directly predicted in silico for a novel chimeric antigen, these structural and epitope-based characteristics provide a rational framework to infer the humoral and cellular immunogenic potential of the designed construct.

In line with this structural and immunogenicity-oriented design, the potential of hcENV to engage ELA-I molecules was further explored through docking analyses involving four peptides included in the construct. These analyses indicated that the selected peptides can be structurally accommodated within the binding grooves of their respective ELA-I molecules. Given the inherent limitations of short-peptide docking into modeled MHC-I structures, these results are presented as complementary structural evidence of peptide–MHC compatibility, rather than as predictors of antigen processing, presentation efficiency, or immunodominance. Nevertheless, the application of peptide–MHC docking as a supportive strategy is well-documented [78,79,80], in contemporary vaccine design, serving here to reinforce the rational framework and the overall immunogenic potential of the proposed hcENV candidate.

As an initial step toward experimental validation, the recombinant expression of the proposed chimeric protein in a bacterial system could be performed to confirm its stability and antigenic recognition by sera from EIAV-infected animals, providing preliminary evidence of expression and epitope accessibility. Given the structural complexity and glycosylation patterns of EIAV envelope proteins, nucleic acid–based platforms (DNA or mRNA vaccines) may also represent suitable alternatives to enable eukaryotic expression and native post-translational processing. Following these proof-of-concept studies, further evaluation in equine cells will be required to assess antigen processing and presentation, including the ability of selected epitopes to stimulate T-cell responses in horses with defined ELA haplotypes, thereby functionally validating the in silico predictions.

## 5. Conclusions

In this work we present the in silico design of a high coverage EIAV vaccine that includes a diversity of conserved epitopes associated with a large number of American field strain variants. This vaccinal candidate contains peptides that can be recognized by several ELA-I and/or ELA-II alleles and potentially induce the secretion of IFN-γ by CD4+ T-cells. Furthermore, four ELA-I epitopes included in the final construct were tested against their ELA alleles in a docking study, were they showed adequate binding. While several predicted B-cell epitopes map to the PND region, which is known to harbor neutralizing determinants, these epitopes are linear and not conformational. Therefore, their actual accessibility and contribution to the induction of neutralizing antibodies remain uncertain.

Although these in silico predictions require experimental validation to confirm their protective efficacy, this design offers a computational framework for developing a vaccine targeting American EIAV strains.

## Figures and Tables

**Table 1:** Comparison of constructs designed with four different linkers. Sequences were compared in terms of their physicochemical properties (residue number, Molecular Weight (MW), Isoelectrical Point (IP)); stability (Instability Index); solubility (GRAVY, CamSol, SoluProt); immunogenicity (Vaxijen 3.0, ANTIGENpro); allergenicity (AllerTOPv.2, AllergenFP) and toxicity (Toxinpred2).

Linker	Fusion Peptide	Residue Number	MW	IP	Instability Index	Aliphatic Index	GRAVY	CamSol	SoluProt	Vaxijen 3.0	ANTIGEN pro	Aller TOPv.2	AllergenFP	Toxinpred2
Criteria for acceptance		-	-	-	<40	>60	<0	>0.80	>0.50	>90%	>0.90	Non allergen	Non allergen	(−)
AAY	-	347	39,351	8.60	42.42	70.09	−0.51	0.19	0.60	100%	0.93	Probable allergen	Non Allergen	(−)
GGGGS	-	361	39,421	8.65	47.57	63.49	−0.58	0.83	0.61	100%	0.97	Non Allergen	Probable allergen	(−)
EAAAK	-	361	40,507	8.60	39.40	69.31	−0.57	0.91	0.59	100%	0.95	Non Allergen	Non Allergen	(−)
GPGPG	+	391	42,822	9.20	37.27	67.14	−0.528	0.65	0.439	100%	0.97	Non Allergen	Non Allergen	(+)
EAAAK	+	392	43,516	8.81	38.92	73.09	−0.449	0.61	0.534	100%	0.95	Non Allergen	Non Allergen	(+)

## Data Availability

The original contributions presented in this study are included in the article/Supplementary Material. Further inquiries can be directed to the corresponding author.

## Supplementary Materials

The following supporting information can be downloaded at: https://www.mdpi.com/article/10.3390/vetsci13030279/s1, Table S1: Results obtained with PROCLEAVE software [42] run on the consensus sequence. Table S2: T-cell epitopes recognized by ELA-I alleles. Table S3: T-cell epitopes recognized by ELA-II alleles. Table S4: High coverage protein design based on overlapping epitope regions connected by three different linkers (AAY, GGGGS, GPGPG and EAAAK). Table S5: Proteasomal cleavage products predicted by iPCPS and their relationship to the EAAAK linker. Table S6: Docking score (DG_cross). Figure S1: Computational confidence metrics for the AlphaFold 3 predicted structure of hcENV. Figure S2: Multiple sequence alignments of each region proposed for inclusion in the vaccine construct. Figure S3: Multiple Sequence Alignment of the PND region [81].

## Abbreviations

The following abbreviations are used in this manuscript:

AA	aminoacid
ADE	Antibody-Dependent Enhancement
BCR	B-cell Receptor
CTL	Cytotoxic T-Lymphocyte
DLV	Donkey Leucocyte Vaccine
DOPE	Discrete Optimized Protein Energy
EIAV	Equine Infectious Anemia Virus
ELA	Equine Lymphocyte Antigen
ELR-1	Equine Lentivirus Receptor-1
ENV	Envelope
gp45	Glycoprotein 45
gp90	Glycoprotein 90
hcENV	High coverage ENV-based chimera
HIV	Human Immunodeficiency Virus
IFN--γ	Gamma interferon
MAFFT	Multiple Alignment using Fast Fourier Transform
MHC	Major Histocompatibility Complex
MSA	Multiple Sequence Alignment
PDB	Protein Data Bank.
PF	Peptide Fusion
PROCLEAVE	Protein Cleave
QMEAN	Qualitative Model Energy Analysis
Th	T helper
UNIPROT	Universal Protein
