# Supplementary material for: A High-Coverage Epitope-Based Vaccine Design for EIAV Envelope Polyprotein Using an Immunoinformatic Approach
Source: Vet Sci. 2026 Mar 17;13(3):279. doi: 10.3390/vetsci13030279 (PMC13030653; doi:10.3390/vetsci13030279)
Supplement: Supplementary file 1 [file vetsci-13-00279-s001.zip › vetsci-4094704-supplementary.pdf]

Table S1

Number	Position	P4-P4' Site	Score	Family	Enzyme name
34984	309	IGGD†SADT	0.998	S01.010	Granzyme B
9868	97	CLAG†TTGG	0.997	C01.060	Cathepsin B
**9869**	**733**	**HLAG†VTGG**	**0.997**	**C01.060**	**Cathepsin B**
20632	500	IVAA†IVAA	0.996	M10.003	Matrix metallopeptidase-2
1795	515	TMSY†VALT	0.987	A01.010	Cathepsin E
20633	476	TPLP†ISSE	0.987	M10.003	Matrix metallopeptidase - 3
20634	843	FPLW†LFWG	0.982	M10.003	Matrix metallopeptidase - 3
20635	14	IPGG†ISTP	0.982	M10.003	Matrix metallopeptidase - 3
48439	276	RVKR†CPID	0.975	S08.073	PCSK-2 peptidase (S08.073)
1796	117	YIGL†VAIG	0.972	A01.010	Cathepsin E
9870	427	QSFG†VIGQ	0.965	C01.060	Catepsine B
**20636**	**733**	**HLAG†VTGG**	**0.962**	**M10.003**	**Matrix metallopeptidase-2**
48440	490	RHKR†DFGI	0.962	S08.073	PCSK-2 peptidase (S08.073)
9871	94	FLLC†LAGT	0.961	C01.060	Cathepsin B
43057	107	WWYE†GLPQ	0.961	S01.269	glutamyl peptidase I (S01.269)
20637	496	GISA†IVAA	0.956	M10.003	Matrix metallopeptidase - 3
20638	297	PPFF†LVNY	0.955	M10.003	Matrix metallopeptidase - 3
**46645**	**490**	**RHKR†DFGI**	**0.955**	**S08.071**	**furin (S08.071)**
**45748**	**490**	**RHKR†DFGI**	**0.949**	**S08.070**	**furin (S08.071)**
6280	682	YLLL†TSSP	0.943	C01.032	cathepsin L (C01.032)

Results obtained with PROCLEAVE software[1] run on the consensus sequence. It predicts potential enzymatic cleavage sites using both sequence and structural information, which let us establish the boundaries of each domain.

[1] F. Li et al., "Procleave: Predicting Protease-specific Substrate Cleavage Sites by Combining Sequence and Structural Information," Genomics. Proteomics Bioinformatics, vol. 18, no. 1, pp. 52–64, Feb. 2020, doi: 10.1016/J.GPB.2019.08.002.

Table S2

T CD8+ EPITOPES	ELA-I Alleles																
	1*01:01	1*02:01	1*02:02	1*03:01	16*01:01	16*03:01	2*01:01	2*01:02	2*01:03	2*03:02	4*01:01	6*01:01	7*01:01	7*02:01	N*01:01	N*05:01	N*06:01
136-GSFPGCRPF-144				■		■						■		■	■		■
142-RPFQNYFSY-150						■	■		■			■		■	■		■
148-FSYETNRSM-156		■		■	■	■	■	■	■	■	■	■	■	■	■	■	■
171-NTATLLEAY-179						■		■		■				■	■		■
177-EAYHREITF-185				■	■	■	■	■	■	■	■	■	■	■	■	■	■
217-RVEDVMNTTEY-227						■		■		■					■		
274-KRCPIDILY-282		■										■		■			
281-LYGIHPIRL-289		■		■								■	■	■			
292-QPPFFLTNF-300							■		■				■	■	■		
417-NYNCVVQSF-425		■	■	■			■	■	■	■		■	■	■	■		
472-TPLPISSEA-480															■		
504-TAIAASATM-512						■									■		■
504-TAIAASAT-511		■		■	■	■	■	■	■	■	■	■	■	■	■	■	■
518-TEVNKIMEV-526	■	■	■	■	■						■	■	■			■	
522-KIMEVQNHTF-531				■	■	■		■		■	■	■	■	■	■	■	■
523-IMEVQNHTF-531				■		■						■	■	■	■		■
529-HTFEVENNTL-538		■		■	■	■		■	■	■	■	■	■	■	■	■	■
640-NTPDSIAQF-648		■		■	■	■		■	■	■		■	■	■	■		■
655-HIANWIPGL-663				■	■	■						■	■		■	■	■
722-DQAQHNIHL-730		■	■	■	■							■	■		■	■	

T-cell epitopes recognized by ELA-I alleles.

Table S3

T CD4+ EPITOPES			PAIRS OF ELA-II ALLELES																												
			DQB1					DQB2																			DQB3	DRB1	DRB2		
			1	2	3	4	5	6	7	1	2	3	4	5	1	2	3	1	2	3	4	5	6	7	8	1	2	3	4	5	6
129-SNAIECWGSFPGCRPFQNYFSYETNRSMHMDNN-161	DQA1	1								■				■									■								
		3					■							■									■								
		4								■				■									■								
		5					■			■				■	■		■	■	■	■		■	■				■				
	DQA2	1			■			■		■				■			■						■								
	DQA3	1								■				■									■								
	DRA	2								■				■	■		■						■								
			1	2	3	4	5	6	7	1	2	3	4	5	1	2	3	1	2	3	4	5	6	7	8	1	2	3	4	5	6
275-CPIDILYGIHPIRLS-289	DQA1	1						■																							
		3																													
		4																													
		5						■																							
	DQA2	1																													
	DQA3	1																													
	DRA	2																													
			1	2	3	4	5	6	7	1	2	3	4	5	1	2	3	1	2	3	4	5	6	7	8	1	2	3	4	5	6
459-ELETWKLVKTSGITP-473	DQA1	1																													
		3																													
		4																													
		5													■		■														
	DQA2	1																													
	DQA3	1																													
	DRA	2																													
			1	2	3	4	5	6	7	1	2	3	4	5	1	2	3	1	2	3	4	5	6	7	8	1	2	3	4	5	6
490-DFGISAIVAAIVAATAIAASATMSYVALTEVNKIMEV-526	DQA1	1								■				■									■								
		3						■		■				■				■					■								
		4								■				■									■								
		5			■		■	■		■				■	■		■	■	■			■	■					■	■		
	DQA2	1						■		■				■				■					■								
	DQA3	1								■				■				■					■								
	DRA	2			■		■	■		■				■	■		■	■					■								
			1	2	3	4	5	6	7	1	2	3	4	5	1	2	3	1	2	3	4	5	6	7	8	1	2	3	4	5	6
620-EILTTLHGARNNLAQSMITFNTPDSIAQF-648	DQA1	1								■				■									■								
		3								■				■									■								
		4								■				■									■								
		5			■		■	■		■				■	■		■	■		■		■	■								
	DQA2	1						■		■				■									■								
	DQA3	1								■				■									■								
	DRA	2						■		■				■				■					■								
			1	2	3	4	5	6	7	1	2	3	4	5	1	2	3	1	2	3	4	5	6	7	8	1	2	3	4	5	6
688-[LRALWKVTSGAGSSGS]-703 725-[QHNIHLAGVTGGSGDK]-740	DQA1	1																													
		3																													
		4																													
		5						■																							
	DQA2	1						■																							
	DQA3	1																													
	DRA	2						■																							

T-cell epitopes recognized by ELA-II alleles.

Table S4

Linker	Protein sequence
AAY	QQSEKAAYSNAIECWGSFPGCRPFQNYFSYETNRSMHMDNNTATLLEAYHREITFAAYLNS<br>SDSSNSVRVEDVMNTTEYAAYTWIPKGCNETWARVKRCPIDILYGIHPIRLSVQPPFFLTNF<br>AAYNYNCVVQSFGVIGQAHLELPRPNKRIRNQSFNQYNCSINNKTELETWKLVKTSGITPLP<br>ISSEANTGLIRHAAYSATMSYVALTEVNKIMEVQNHTFEVENNTLAAYDILTTLHAARNNL<br>AQSMITFNTPDSIAQFGKDLWSHIANWIPGLAAYLRALWKVTSGAGSSGSRYLKKKFHHKH<br>ASREDTWDQAQHNIHLAGVTGGSGDKYYKQKYSRNDWNGE
GGGGS	QQSEKGGGGSSNAIECWGSFPGCRPFQNYFSYETNRSMHMDNNTATLLEAYHREITFGGG<br>GSLNSSDSSNSVRVEDVMNTTEYGGGGSTWIPKGCNETWARVKRCPIDILYGIHPIRLSVQP<br>PFFLTNFGGGGSNYNCVVQSFGVIGQAHLELPRPNKRIRNQSFNQYNCSINNKTELETWKL<br>VKTSGITPLPISSEANTGLIRHGGGGSSATMSYVALTEVNKIMEVQNHTFEVENNTLGGGGS<br>DILTTLHAARNNLAQSMITFNTPDSIAQFGKDLWSHIANWIPGLGGGGSLRALWKVTSGAG<br>SSGSRYLKKKFHHKHASREDTWDQAQHNIHLAGVTGGSGDKYYKQKYSRNDWNGE
EAAAK	QQSEKEAAAKSNAIECWGSFPGCRPFQNYFSYETNRSMHMDNNTATLLEAYHREITFEAAA<br>KLNSSDSSNSVRVEDVMNTTEYEAAAKTWIPKGCNETWARVKRCPIDILYGIHPIRLSVQPP<br>FFLTNFEAAAKNYNCVVQSFGVIGQAHLELPRPNKRIRNQSFNQYNCSINNKTELETWKLV<br>KTSGITPLPISSEANTGLIRHEAAAKSATMSYVALTEVNKIMEVQNHTFEVENNTLEAAAKD<br>ILTTLHAARNNLAQSMITFNTPDSIAQFGKDLWSHIANWIPGLEAAAKLRALWKVTSGAGS<br>SGSRYLKKKFHHKHASREDTWDQAQHNIHLAGVTGGSGDKYYKQKYSRNDWNGE
	SNAIECWGSFPGCRPFQNYFSYETNRSMHMDNNTATLLEAYHREITFEAAAKLNSSDSSNS<br>VRVEDVMNTTEYEAAAKARVKRCPIDILYGIHPIRLSVQPPFFLTNFEAAAKNYNCVVQSFG<br>VIGQAHLELPRPNKRIRNQSFNQYNCSINNKTELETWKLVKTSGITPLPISSEANTGLIRHEA<br>AAKDFGISAIVAAIVAATAIAASATMSYVALTEVNKIMEVQNHTFEVENNTLEAAAKDILTT<br>LHAARNNLAQSMITFNTPDSIAQFGKDLWSHIANWIPGLEAAAKLRALWKVTSGAGSSGSR<br>YLKKKFHHKHASREDTWDQAQHNIHLAGVTGGSGDKYYKQKYSRNDWNGEEAAAKQQS<br>EKEAAAKTWIPKGCNETWARVKRCP
GPGPG	SNAIECWGSFPGCRPFQNYFSYETNRSMHMDNNTATLLEAYHREITFGPGPGLNSSDSSNSV<br>RVEDVMNTTEYGPGPGARVKRCPIDILYGIHPIRLSVQPPFFLTNFGPGPGNYNCVVQSFGVI<br>GQAHLELPRPNKRIRNQSFNQYNCSINNKTELETWKLVKTSGITPLPISSEANTGLIRHGPGP<br>GDILTTLHAARNNLAQSMITFNTPDSIAQFGKDLWSHIANWIPGLGPGPGLRALWKVTSGA<br>GSSGSRYLKKKFHHKHASREDTWDQAQHNIHLAGVTGGSGDKYYKQKYSRNDWNGEGP<br>GPGDFGISAIVAAIVAATAIAASATMSYVALTEVNKIMEVQNHTFEVENNTLGPGPGQQSEK<br>KKTWIPKGCNETWARVKRCPKK

High coverage protein design based on overlapping epitope regions connected by three different linkers (AAY, GGGGS, GPGPG and EAAAK). The marked sequence (EAAAK linker) was selected as the final high coverage Env vaccinal construct (hcENV).

Table S5

Peptide	Position	ImPrt.Int.Cleavage[1]	ImPrt. C-term Clevage[1]
AAKNYNCVV	132-140	⊘	✓
KQKYSRNDW	350-358	⊘	✓
SATMSYVAL	211-219	⊘	✓
GCNETWARV	94-102	⊘	✓
ITFEAAAKL	55-63	⊘	✓
MDNNTATLL	40-48	⊘	✓
CSINNKTEL	170-178	⊘	✓
TFEVENNTL	232-240	⊘	✓
HIANWIPGL	281-289	⊘	✓
QHNIHLAGV	332-340	⊘	✓
NSSDSSNSV	64-72	⊘	✓
QSEKEAAAK	2-10	⊘	✓
LIRHEAAAK	202-210	⊘	✓
AGSSGSRYL	305-313	⊘	✓
TTEYEAAAK	80-88	⊘	✓
QYNCSINNK	167-175	⊘	✓
IAQFGKDLW	271-279	⊘	✓
AKSATMSYV	209-217	⊘	✓
NTPDSIAQF	266-274	⊘	✓
ISSEANTGL	194-202	⊘	✓
AKSNAIECW	9-17	⊘	✓
TLHAARNNL	250-258	⊘	✓
DQAQHNIHL	329-337	⊘	✓
GDKYYKQKY	345-353	⊘	✓
GSFPGCRPF	18-26	⊘	✓
YETNRSMHM	32-40	⊘	✓
AAAKTWIPK	85-93	⊘	✓
RPFQNYFSY	24-32	⊘	✓
EDVMNTTEY	75-83	⊘	✓

Peptide	Position	ImPrt.Int.C leavage[1]	ImPrt. C-term Clevage[1]
GAGSSGSRY	304-312	🛇	✔
SIAQFGKDL	270-278	🛇	✔
NAIECWGSF	12-20	🛇	✔
NFEAAAKNY	128-136	🛇	✔
IRNQSFNQY	160-168	🛇	✔
GCRPFQNYF	22-30	🛇	✔
YEAAAKTWI	83-91	🛇	✔
LWSHIANWI	278-286	🛇	✔
HMDNNTATL	39-47	🛇	✔
ARNNLAQSM	254-262	🛇	✔
ITFNTPDSI	263-271	🛇	✔
FGKDLWSHI	274-282	🛇	✔
AAAKNYNCV	131-139	🛇	✔
GGSGDKYYK	342-350	🛇	✔
PGCRPFQNY	21-29	🛇	✔
PDSIAQFGK	268-276	🛇	✔
RNNLAQSMI	255-263	🛇	✔
SSEANTGLI	195-203	🛇	✔
EAYHREITF	49-57	🛇	✔
GSRYLKKKF	309-317	🛇	✔
EAAAKSNAI	6-14	🛇	✔
KHASREDTW	320-328	🛇	✔
EITFEAAAK	54-62	🛇	✔
EAAAKSATM	206-214	🛇	✔
KKKFHHKHA	314-322	🛇	✔
TWDQAQHNI	327-335	🛇	✔
IPKGCNETW	91-99	🛇	✔
TGGSGDKYY	341-349	🛇	✔
NYFSYETNR	28-36	🛇	✔
SGDKYYKQK	344-352	🛇	✔

Peptide	Position	ImPrt.Int.Cleavage[1]	ImPrt. C-term Clevage[1]
YLKKKFHHK	312-320	⊘	✓
AAKSATMSY	208-216	⊘	✓
FSYETNRSM	30-38	⊘	✓
EYEAAAKTW	82-90	⊘	✓
DLWSHIANW	277-285	⊘	✓
PKGCNETWA	92-100	⊘	✓
GSSGSRYLK	306-314	⊘	✓
SSGSRYLKK	307-315	⊘	✓
QQSEKEAAA	1-9	⊘	✓
REITFEAAA	53-61	⊘	✓
NTTEYEAAA	79-87	⊘	✓
GLIRHEAAA	201-209	⊘	✓
LHAARNNLA	251-259	⊘	✓
TFNTPDSIA	264-272	⊘	✓
SREDTWDQA	323-331	⊘	✓
NKRIRNQSF	157-165	⊘	✓
NLAQSMITF	257-265	⊘	✓
ECWGSFPGC	15-23	⊘	✓
SGAGSSGSR	303-311	⊘	✓
SGSRYLKKK	308-316	⊘	✓
SFNQYNCSI	164-172	⊘	✓
CWGSFPGCR	16-24	⊘	✓
SMHMDNNTA	37-45	⊘	✓
RHEAAAKSA	204-212	⊘	✓
GKDLWSHIA	275-283	⊘	✓
NQSFNQYNC	162-170	⊘	✓
QAHNIHLA	330-338	⊘	✓

Proteasomal cleavage products predicted by iPCPS and their relationship to the EAAAK linker: Proteasomal cleavage predictions were obtained using iPCPS with the immunoproteasome model (Model 1), peptide mode (9 amino acids). All predicted peptides lacking internal cleavage sites are shown. The presence of the EAAAK linker sequence within or overlapping each peptide was manually inspected. What is highlighted in the box are the sequences corresponding to the CTLs epitopes in our vaccine sequence.

(1) <u>Interpretacion:</u> Im Prt. C- term Clevage (**I**mmuno**pro**teasome **C-term**inal **Cleavage),** and <u>ImPrt.Int</u>. Cleavage (**I**mmuno**pro**teasome **Int**ernal **Cleavage)**: Both signals (;) allow for the selection of peptides predicted to bind to MHC-I molecules, which are likely to result from C-terminal proteosomal cleavage without internal degradation.

Table-S6

Score[1]	ELA-allele	Protein	Epitope	dG cross[2]	dSASA hphobic(Å2)	dSASA int(Å2)	dSASA polar(Å2)	Hbonds unsat	Hbond E_fraction
0.03240	Eqca-1*01:01	gp90	**TEVNKIMEV**	-2.751	880.224	1583.881	703.658	7	0.2
0.01475	Eqca-7*02:01	gp90	**RPFQNYFSY**	-3.141	1183.671	1992.398	808.728	2	0.233
0.03780	Eqca-7*02:01	gp90	**EAYHREITF**	-3.166	1174.465	1949.422	774.956	15	0.291
0.01700	Eqca-2*03:02	gp45	**NTPDSIAQF**	-3.270	710.003	1179.895	469.891	8	0.186
**References and Controls**									
0.18000	Eqca-N*06:01	gp90	**RVEDTNTTEY**	-2.308	848.9	1751.597	902.697	17	
**1.00850**	Eqca-7*02:01	gp90	**TGIYQVPIF[3]**	**-2.398**	1073.433	1821.326	747.893	24	0.173
	Eqca-N*06:02	gp90	**RVEDVTNTAEYW[4]**	-3.509	1057.002	1898.203	841.201	8	0.279

Docking score (DG_cross), solvent accessible surface of the complex interface (dSASA_int), discriminated by its hydrophobic (dSASA_hphobic) and polar contribution (dSASA_polar) for each test. Also included in the table are hydrogen bonds formed at the complex interface that are available but not formed (unsatHbonds). Superscript means: 1) MHC-Flurry Percentil Score 2) dG normalized by dSASAx100 3) low percentil affinity for Eqca-7*02:01 4) Experimental Solved Complex (4ZUV).

Figure S1

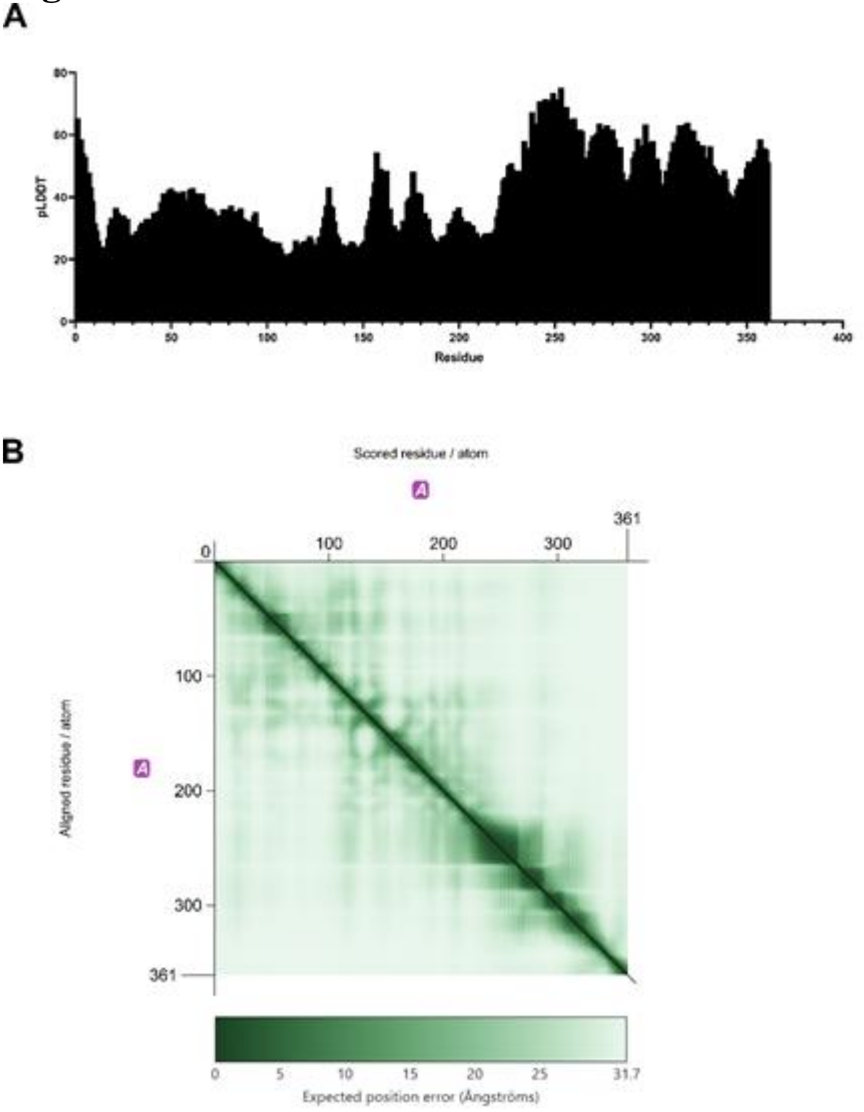

Computational confidence metrics for the AlphaFold 3 predicted structure of hcENV. A) Per-residue pLDDT (predicted Local Distance Difference Test) scores plotted along the primary sequence. B) Predicted Aligned Error (PAE) matrix indicating the confidence in the relative orientation of domains and subunits.

Data availability
The structural models generated by AlphaFold 3 in this study are available at Zenodo (doi: 10.5281/zenodo.18322271).

Figure S2

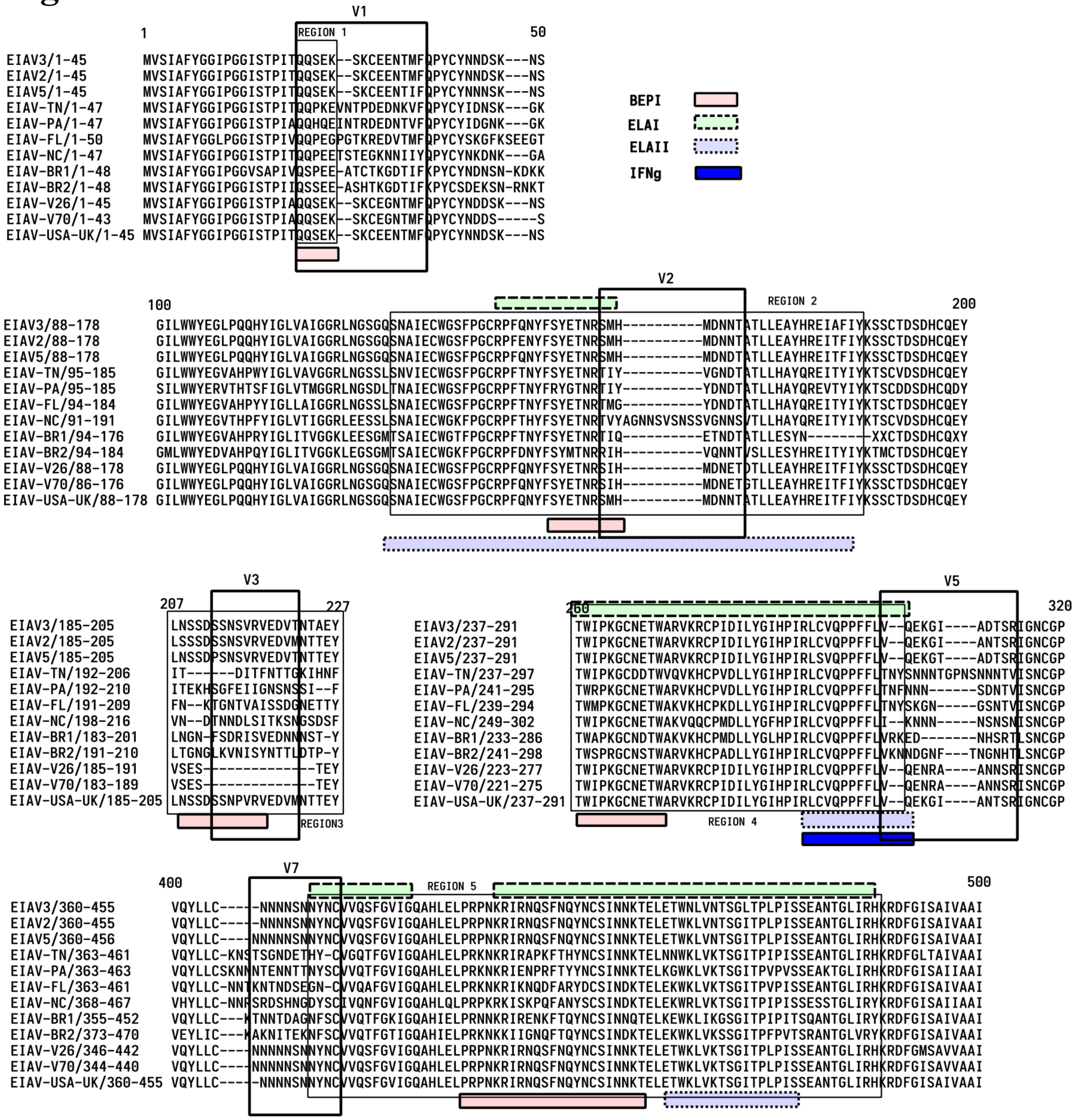

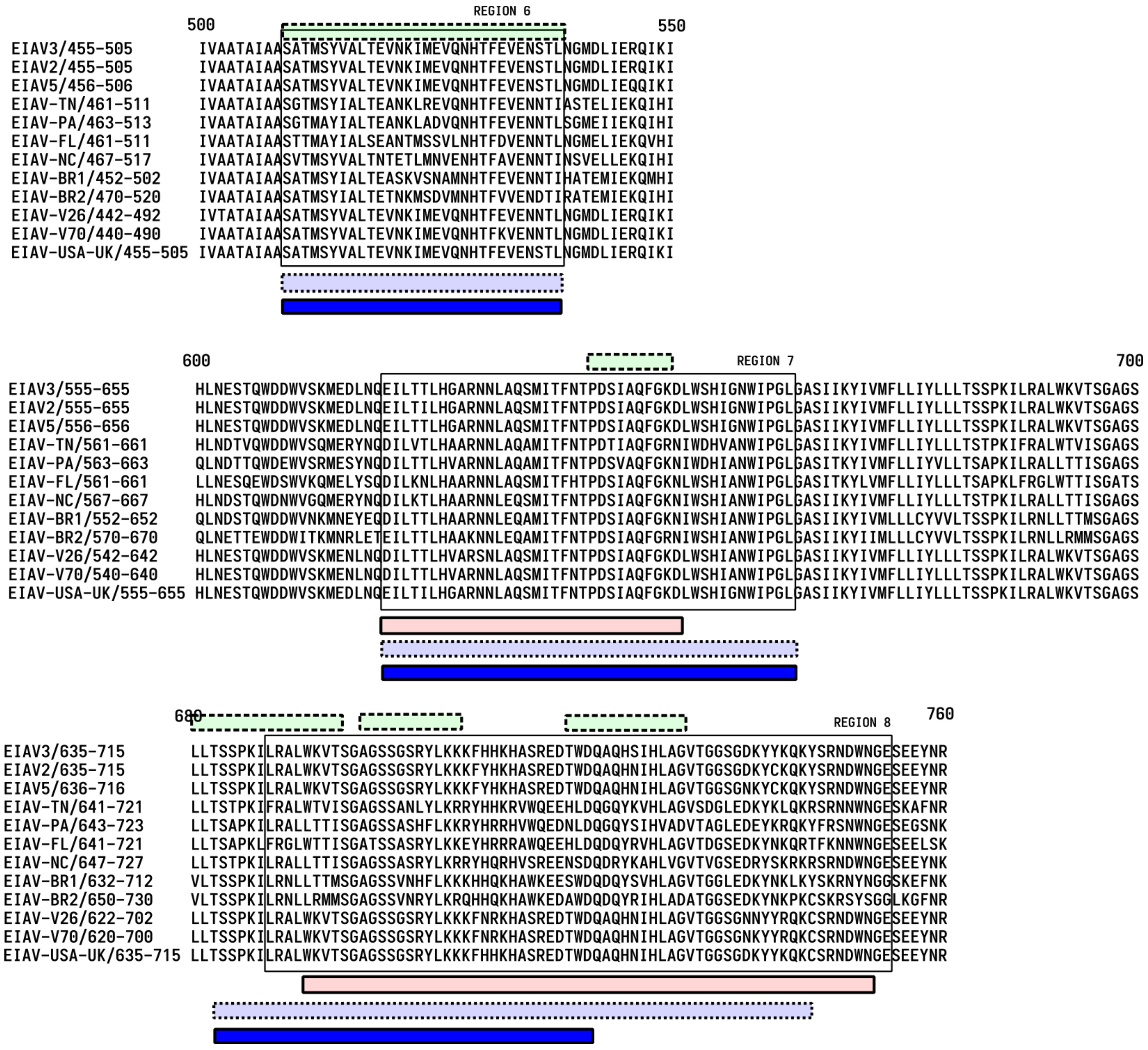

Multiple sequence alignments of each region proposed for inclusion in the vaccine construct. Below the alignment, residues corresponding to B-cell epitopes predicted by BEPI2 are indicated by solid-line squares with a red background. Above the alignment, T-cell epitopes predicted by ELAI (MHCflurry 2.0) are shown as dashed-line rectangles with a green background. Below the alignment, epitopes predicted as IFN-γ inducers are indicated by solid-line squares with a blue background. In addition, MHC class II epitopes predicted by NetMHCpan 4.1 (ELAI I) are shown below the alignment as dotted-line rectangles with a light-blue background. Black rectangles indicate the selected regions as well as the previously described variable regions of this virus.

Figure S3

```
EIAV3/178-208          YQCKKVNLNSSDSSNSVRVEDVTNTAEYWGF
EIAV2/178-208          YQCKKVNLSSSDSSNSVRVEDVMNTTEYWGF
EIAV5/178-208          YQCKQVNLNSSDPSNSVRVEDVTNTTEYWGF
EIAV-TN/185-209        YKCKNITIT------DITFNTTGKIHNFQKF
EIAV-PA/185-213        YQCKQVNITEKHSGFEIIGNSNSSI--FWGF
EIAV-FL/184-212        YRCKQVRFN--KTGNTVAISSDGNETTYWGF
EIAV-NC/191-219        YVCQKVKVN--DTNNDLSITKSNGSDSFWKF
EIAV-BR1/176-204       YECERVRLNGN-FSDRISVEDNNNST-YWDF
EIAV-BR2/184-213       YECRNVQLTGNGLKVNISYNTTLDTP-YWNF
EIAV-V26/178-194       YQCQKVNVSES--------------TEYWGF
EIAV-V70/176-192       YQCQKVNVSES--------------TEYWGF
EIAV-USA-UK/178-208    YQCKKVNLNSSDSSNPVRVEDVMNTTEYWGF
```

Multiple Sequence Alignment of the PND region (Principal Neutralizing Domain) using the 12 American sequences analyzed in this study. Asparagine residues within the consensus sequon are highlighted in orange as potentially N-glycosylated sites (NetNGlyc-1.0 score ≥ 0.6 (Gupta R, Brunak S. Prediction of glycosylation across the human proteome and the correlation to protein function. Pac Symp Biocomput. 2002;:310-22. PMID: 11928486)).
